# Captivity Shifts Gut Microbiota Communities in Plateau Zokor (*Eospalax baileyi*)

**DOI:** 10.3390/microorganisms12040789

**Published:** 2024-04-12

**Authors:** Daoxin Liu, Bin Li, Pengfei Song, Feng Jiang, Tongzuo Zhang

**Affiliations:** 1 College of Agriculture and Animal Husbandry, Qinghai University, Xining 810016, China; liudx@qhu.edu.cn; 2 Key Laboratory of Adaptation and Evolution of Plateau Biota, Northwest Institute of Plateau Biology, Chinese Academy of Sciences, Xining 810001, China; libin@nwipb.cas.cn (B.L.); pfsong@nwipb.cas.cn (P.S.); jiangfeng@nwipb.cas.cn (F.J.); 3 Qinghai Provincial Key Laboratory of Animal Ecological Genomics, Xining 810001, China

**Keywords:** plateau zokor, gut microbiota, captivity, assembly process

## Abstract

The gut microbiota in animals is a dynamic ecosystem influenced by both the host itself and the environment it inhabits. It is known that short-term captivity can significantly impact the gut microbiota of plateau zokors, leading to substantial inter-individual variation. However, the specific changes in the assembly process of the gut microbiota in plateau zokors during captivity remain unclear. In this study, we conducted a comparative analysis on the assembly process of the gut microbiota in 22 male plateau zokors from the same location in Qinglin Township, Datong County, Qinghai Province, before (W) and after (L) laboratory rearing. We performed a single-factor correlation network analysis on the top 50 genera with relative abundance in each group. The results revealed that captivity increased the complexity of the gut microbiota in plateau zokors, indicating a higher number of interactions between different microbial species. However, this increase in complexity was accompanied by a decrease in stability, suggesting a higher degree of variability and potential disruption in the microbial community. According to the results of the neutral community model, the gut microbiota of plateau zokors in the W had a higher Nm value (Nm = 48,135) compared to the L (Nm = 39,671), indicating that species dispersal of the gut microbiota was greater in the wild than in captivity. In the wild, the modified stochasticity ratio (MST) was less than 0.5, suggesting that deterministic processes dominated. However, after 15 days of laboratory rearing, the MST became greater than 0.5, indicating a shift toward stochastic processes, and this difference was highly significant (*p* < 0.001). This differs from research related to aboveground animals. This study provides theoretical support for the application of gut microbiota in subterranean endangered species.

## 1. Introduction

The plateau zokor (*Eospalax baileyi*) is a typical subterranean herbivorous animal that resides in the Qinghai–Tibet Plateau, spending most of its life in dark and damp underground burrows where most of its activities, including foraging and reproduction, take place [1,2,3]. They are typically solitary except during the breeding season [4]. The plateau zokor primarily feeds on plant roots, and due to the influence of underground burrows and food scarcity, it has even developed a preference for some toxic plants, such as *Stellera chamaejasme* [5]. When the grasslands become degraded, the population density of plateau zokors significantly increases. This high density of plateau zokors has a negative impact on the grassland ecosystem. As a result, plateau zokors are considered pests [6]. Benefiting from all the characteristics described above, the plateau zokor is increasingly attracting the attention of researchers as an ideal model for studying high-altitude adaptation, cold adaptation, and detoxification mechanisms [5,7].

Gut microbiota is a complex ecological community and plays an essential role in host nutrition, metabolism, immunity, and health [8,9]. While the gut microbiota modulates host physiology and metabolism through different mechanisms, the composition and function of the gut microbiota are strictly linked to the host and its environment [10,11]. Numerous studies have demonstrated that captivity could result in significant changes in the gut microbiota of wildlife. This is primarily attributed to the need for these animals to adapt to changes in their living conditions and dietary patterns when they are relocated from their natural habitats to captivity [12,13,14]. A lack of a robust and native microbial community is considered to be a significant contributing factor to the poor health of animals in captivity and the low success rate of certain reintroduction programs [15,16]. Thus, there is an urgent need to address whether there is a consistent pattern of gut microbiota variation between wild animals and captive animals [17]. However, most previous studies on gut microbiota in wildlife have primarily focused on the composition, structure, and function of the gut microbiota, with less attention given to the impact of captivity on the assembly process of gut microbiota [13,18]. Niche-based theories and neutral-based theories constitute two important and complementary mechanisms for understanding microbial community assemblies. Neutral theory suggests that random processes, such as birth, death, migration, speciation, and limited dispersal, shape microbial community structure, assuming a random equilibrium between microbial loss and gain within a community. Niche theory posits that microbial communities are determined by deterministic abiotic factors (environmental factors such as pH and temperature) and biotic factors (species interactions such as competition and predation), causing different microbial habitat preferences and levels of adaptability [19].

The limited researches on terrestrial animals have shown that captivity shifts the ecological assembly process of gut microbiota of white-lipped deer (*Cervus albirostris*) by raising the contribution of deterministic processes [14], and captivity would significantly reduce the contribution of stochastic processes while the plateau pikas (*Ochotona curzoniae*) were captured and kept for a duration of 4 months [20]. However, it is not yet clear whether the gut microbiota assembly process of wildlife would be altered during short-term artificial captivity and whether subterranean animals and aboveground animals would exhibit the same trend. Our previous research has shown that short-term captivity can significantly influence their gut microbiota and lead to greater individual differences [13]. However, it is unclear how the assembly process of the gut microbiota in plateau zokors changes in captivity.

In this study, we conducted a comparative analysis of the correlation network and assembly process of gut microbiota in 22 male plateau zokors collected from the same location in Qinglin Township, Datong County, Qinghai Province, before and after laboratory rearing. Our goal was to address the following two questions: (1) Does short-term artificial rearing affect the correlation network and assembly process of the plateau zokor’s gut microbiota? (2) If it does, is this effect consistent with changes observed in surface-dwelling animals?

## 2. Materials and Methods

### 2.1. Data Collection

Sequence data are available from Sequence Read Archive (SRA) BioProject PRJNA690964 which had been uploaded by us previously [13]. In this dataset, 22 individual plateau zokors were captured alive on the same day in May 2019 in Qinglin Township, Datong County, Qinghai Province. (37°8′20″ N, 101°15′1″ E, altitude 3111 m). After capturing the plateau zokors, the first fresh fecal samples were collected from each individual and labeled as W01 to W22 (W group). In the laboratory, each plateau zokor was individually housed in a stainless-steel box that had been disinfected with alcohol (40 cm × 30 cm × 25 cm, wood shavings were used as bedding material, and the windows were shaded with shade curtains) and fed exclusively with carrots as the sole food source during the experimental period. They were kept in a dark environment for 15 days. Subsequently, fresh fecal samples were collected again from each individual before and after laboratory rearing, labeled as L01 to L22 (L group). The experimental design and procedures were approved by the Animal Care and Use Committee of Northwest Institute of Plateau Biology, Chinese Academy of Sciences (IACUC Issue No. NWIPB2019009).

### 2.2. Data Processing

Samples were subjected to DNA extraction using the cetyltrimethylammonium bromide (CTAB) method. The concentration and purity of the extracted DNA were assessed on 1% agarose gels and then diluted to a concentration of 1 ng/µL with sterile water. Subsequently, a specific region (V3–V4) of the 16S rRNA gene from fecal microbiota was amplified by polymerase chain reaction (PCR) using the primer pair 341F-806R (341F: 5′-CCTAYGGGRBGCASCAG-3′, 806R: 5′-GGACTACNNGGGTATCTAAT-3′) along with barcodes. Each PCR reaction mixture contained 15 µL of Phusion^®^ High-Fidelity PCR Master Mix (New England Biolabs, Ipswich, MA, USA), 0.2 µM of each primer, and 10 ng of the target DNA. The cycling conditions consisted of an initial denaturation step at 98 °C for 1 min, followed by 30 cycles of denaturation at 98 °C for 10 s, annealing at 50 °C for 30 s, extension at 72 °C for 30 s, and a final extension at 72 °C for 5 min. The PCR products were visualized on a 2% agarose gel containing SYB green. Afterward, the PCR products were mixed in equal proportions, and purification was performed using the Qiagen Gel Extraction Kit (Qiagen, Hilden, Germany). The sequencing libraries were prepared using the NEBNext^®^ Ultra™ II DNA Library Prep Kit (cat no. E7645), and their quality was assessed using a Qubit@ 2.0 fluorometer (Thermo Scientific, Waltham, MA, USA) on the Agilent (Santa Clara, CA, USA) Bioanalyzer 2100 system.

Using the fastp software (version 0.20.0) [21] for quality control of raw sequences and FLASH software (version 1.2.7) [22] for merging, the steps taken were as follows: Filter bases with quality values below 20 at the end of reads. Set a 50 bp window, and trim bases from the end of the window if the average quality value within the window is below 20. Filter out reads that are less than 50 bp after quality control, and remove reads containing N bases. Based on the overlap relationship between paired-end reads, merge paired reads into a single sequence with a minimum overlap length of 10 bp. Allow a maximum mismatch rate of 0.2 within the overlap region of merged sequences, and filter out sequences that do not meet this criterion. Differentiate samples based on the barcode and primer sequences at the ends of the sequences. Adjust the sequence orientation accordingly. Allow a maximum of 0 mismatches in barcodes and a maximum of 2 mismatches in primer sequences. Using UCHIME (version 4.2) [23], perform OTU clustering on the sequences based on a 97% similarity threshold [24], and remove chimeras. Remove OTUs that are only present in one sample and have a total sequence count of less than 5. For each OTU, select a representative sequence for further annotation. Utilize the Silva 16S rRNA database (version 138) [25] and the Mothur software (version 1.31.2) [26] for taxonomic annotation of each OTU’s representative sequence, using a confidence threshold of 0.8. Simultaneously, eliminate OTUs with annotation results as chloroplasts and mitochondria.

Network analysis can be used to analyze the correlation between microbial communities [27]. In the network graph, the nodes represent species-level nodes. By calculating the node degree distribution, node connectivity (degree), and other attributes of the network, the correlation between microbial communities can be obtained, providing a comprehensive reflection of the data information. Species with a total abundance in the top 50 and an absolute correlation coefficient ≥ 0.6 (*p* < 0.05) were selected to construct a genus-level single-factor correlation network. The correlation network analysis was conducted using the diversity cloud analysis platform (www.majorbio.com accessed on 1 June 2023) by Shanghai Majorbio Pharmaceutical Technology Co., Ltd., (Shanghai, China). 

In order to assess the impact of captivity on the gut microbiota ecological assembly process in plateau zokors, we utilized the neutral community model (NCM) by using minpack.lm (version 1.2-1), Hmisc (version 4.4-0), and stats4 (version 3.5.3) packages to quantify the importance of neutral processes (random processes) [28,29,30]. Additionally, we employed the modified stochasticity ratio (MST) by using NST packages (version 3.0.6) to reflect the contributions of stochastic and non-stochastic assembly processes [31]. The MST is an extension based on the beta diversity index, used to measure the relative position of observed values between pure deterministic and pure random assembly, reflecting the contribution of random assembly to deterministic assembly [28]. The randomness of ecological processes in the gut microbiota was quantified by comparing the values between the two groups. These analyses were performed using R (version 4.1.1).

## 3. Results

### 3.1. Data Profiling

This study obtained a total of 3,921,111 raw data sequences, with an average of 89,116 ± 6389 reads per sample. Among these, 3,643,113 sequences were successfully assembled, accounting for 92.91% of the total. After quality control, a total of 3,240,868 effective sequences were obtained from 44 samples, with an average effectiveness rate of 88.96%. The average length of each sequence was 418.53 base pairs, and the Q20 and Q30 values were 97.96% and 93.78%, respectively. To facilitate uniform analysis, this study down sampled all samples to a minimum sequence number of 59,896.

### 3.2. Correlation Network Analysis

**Figure 1:**
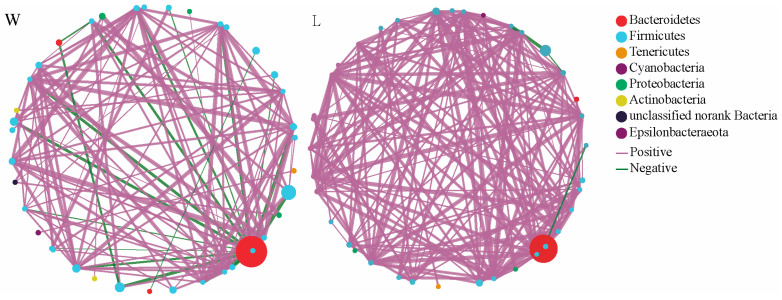
Correlation network analysis. W represents the wild group, while L represents the captive group. The size and color of the nodes represent the relative abundance of the gut microbiota and heritability estimates, respectively. The solid lines in purple and the solid lines in green represent positive and negative correlations, respectively. The width of the lines represents the strength of the correlation.

In Figure 1, the node size represents the species abundance, and the color of the links represents positive or negative correlations, with purple indicating positive correlations and green indicating negative correlations. The thickness and number of lines represent the correlation between species. The results showed that the W group had 39 nodes, with 28 nodes belonging to the Firmicutes phylum, accounting for 71.79%. There were 121 edges, with 101 positive correlations accounting for 83.47% and 20 negative correlations accounting for 16.53%. On the other hand, the L group had 38 nodes, with 30 nodes belonging to the Firmicutes phylum, accounting for 78.95%. There were 172 edges, with 169 positive correlations accounting for 98.26% and 3 negative correlations accounting for 1.74%. This indicates that the gut microbiota network in the L group is more complex, with tighter connections between microorganisms compared to the W group.

### 3.3. Community Assembly of Gut Microbiota

**Figure 2:**
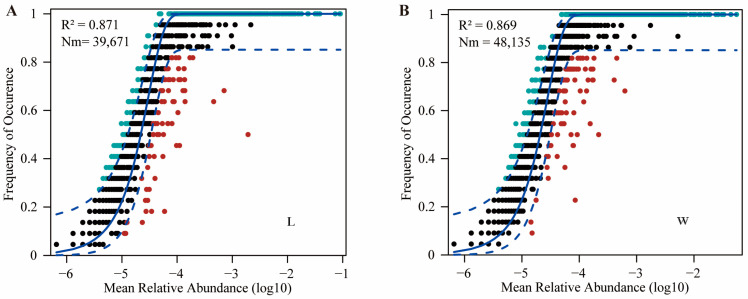
The neutral community model. (**A**) represents the wild group, while (**B**) represents the captive group. The solid line represents the fit of the neutral community model, while the upper and lower dashed lines represent the 95% confidence interval of the model predictions. R^2^ represents the overall goodness of fit of the neutral community model. Nm is the product of community size (N) and migration rate (m), quantifying the estimated diffusion between communities and determining the correlation between occurrence frequency and relative abundance in the region.

The neutral community model (NCM) quantifies the importance of neutral processes (Figure 2). The results showed that the NCM successfully estimated the relationship between the occurrence frequency of OTUs and their relative abundance changes, with high explanatory power in both field conditions and indoor rearing processes (R^2^ > 0.85). This indicates that random processes are crucial for the assembly of gut microbiota communities in plateau zokors. Additionally, the Nm values (Nm = 48,135) of gut microbiota in the W group were higher than those in the L group (Nm = 39,671), suggesting a higher level of species dispersal in the wild for plateau zokor gut microbiota compared to captivity.

**Figure 3:**
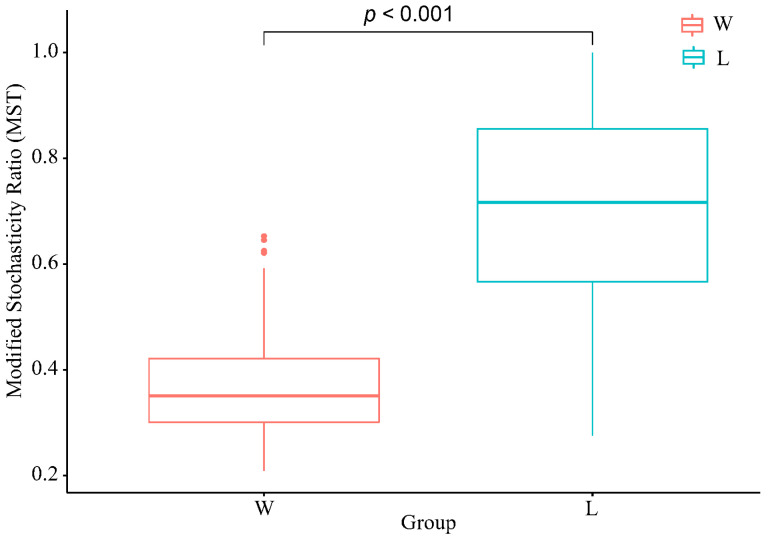
The modified stochasticity ratio. W represents the wild group, while L represents the captive group.

The Modified Stochasticity Ratio (MST) was used to quantify the stochasticity in ecological processes (Figure 3). The results showed that the MST values of the W group were mostly below the threshold line of 0.5, indicating a dominance of deterministic processes. On the other hand, the majority of the L group had MST values above the threshold line, indicating a dominance of stochastic processes. There was a significant difference in MST values between the two groups (*p* < 0.01), indicating a significant difference in the impact of stochastic processes on the two communities.

## 4. Discussion

### 4.1. Captivity Affects the Stability of the Gut Microbiota in Plateau Zokors

Alpha diversity can be used to assess the impact of specific factors on the species richness and evenness of gut microbiota. Beta diversity is used to evaluate the similarity of communities compared to other analyzed samples [32]. The previous studies on captive animal microbiomes have documented that captivity generally reduces alpha diversity of gut microbiomes and also the effects on microbial beta diversity [33,34]. Our previous study on the effect of short-term captivity on the gut microbiota of plateau zokors confirmed that both alpha and beta diversity of the gut microbiota changes after a brief period of captivity [13]. The stability of gut microbiota is deemed essential for the health and well-being of the host. This is because it guarantees the preservation of beneficial symbionts and their correlated functions over time [35]. In addition, the complexity of the interactions visualized through ecological networks could potentially indicate the stability of the ecosystem [36]. In this study, the results of the univariate correlation network analysis showed that after 15 days of captivity, the gut microbiota network of plateau zokors became more complex than that of the W group, with a significant increase in the proportion of positive correlations and a notable decrease in negative correlations. The increase in the proportion of positive correlations can create dependency and the potential for mutual downfall [37]. This indicates that after captivity, the complexity of the gut microbiota in plateau zokors increases, while the stability decreases. The inability to successfully breed plateau zokors in the laboratory at present [3] may be due in part to this reason.

### 4.2. Captivity Affects the Assembly Process of the Gut Microbiota in Plateau Zokors

When wild animals enter artificial environments, they undergo rapid changes in their living conditions and transition from natural food sources to diets that are less diverse or compositionally different [38]. These changes can lead to alterations in the composition and assembly process of their gut microbiota. Zhang et al.’s study showed that captive breeding significantly reduced the importance of stochastic processes in the assembly of gut microbial communities in plateau pikas [20]. Similarly, Li et al.’s research revealed that captivity altered the ecological assembly process of gut microbiota in white-lipped deer by increasing the contribution of deterministic processes [14]. In this study, the neutral model provided a better explanation for the changes in bacterial communities, indicating the significant role of stochastic processes in the assembly of gut microbial communities in both wild and captive groups. The Nm of the W group was higher than that of the L group, suggesting intergroup differences in the species dispersal ability of gut microbiota in plateau zokors, with a higher dispersal limitation in the L group. The one-animal-per-cage rearing method used in this study effectively prevented the dispersal of gut microbiota between individuals, indirectly suggesting that plateau zokors may not lead entirely solitary lives in the wild. It is worth noting that this study focused on male individuals, thus further research is needed to explore the mating system and mechanisms of interpersonal communication among plateau zokors. Additionally, plateau zokors exhibit coprophagy [11], which may also contribute to the dispersal of gut microbiota.

To further validate the results of the neutral model, this study employed a corrected randomization approach to assess the relative importance of deterministic and stochastic processes in community assembly. The results showed that the W group was primarily dominated by deterministic processes, while the L group was dominated by stochastic processes. These results are inconsistent with studies on aboveground animals. There are three possible reasons for this discrepancy. Firstly, as a typical subterranean rodent species endemic to the Qinghai–Tibetan Plateau, plateau zokors spend the majority of their lives in underground burrows [39]. Compared to aboveground animals, zokors’ living environments and temperatures are relatively stable, and they face lower predation risks [40,41]. Secondly, the zokors in this study were only kept in captivity for 15 days, which is a relatively short period. It would be worth investigating whether the assembly process of their gut microbiota changes with longer periods of captivity. Thirdly, in contrast to wild environments, the captive zokors were provided with only carrots as their food. Tang et al.’s research found that dietary fiber content can influence the assembly process of gut microbiota, with high-fiber diets being dominated by deterministic processes [42]. In the wild, plateau zokors are dietary generalists, consuming up to 66 plant species, mainly feeding on their roots and rhizomes [43], which have relatively high fiber content.

## 5. Conclusions

In this study, we compared the gut microbial networks and assembly processes of plateau zokors before and after a short period of captivity. We observed an increase in complexity and a decrease in stability of their gut microbiota following capture. Furthermore, the assembly process shifted from being primarily deterministic processes in the wild to being predominantly stochastic processes after capture. This study provides theoretical support for the application of gut microbiota in subterranean endangered species.

## Data Availability

Sequence data are available from Sequence Read Archive (SRA) BioProject PRJNA749684.
